# Effect of Ag-Decorated BiVO_4_ on Photoelectrochemical Water Splitting: An X-ray Absorption Spectroscopic Investigation

**DOI:** 10.3390/nano12203659

**Published:** 2022-10-18

**Authors:** Ta Thi Thuy Nga, Yu-Cheng Huang, Jeng-Lung Chen, Chi-Liang Chen, Bi-Hsuan Lin, Ping-Hung Yeh, Chao-Hung Du, Jau-Wern Chiou, Way-Faung Pong, K. Thanigai Arul, Chung-Li Dong, Wu-Ching Chou

**Affiliations:** 1 Department of Electrophysics, National Yang Ming Chiao Tung University, Hsinchu 30010, Taiwan; 2 Research Center for X-ray Science & Department of Physics, Tamkang University, New Taipei City 25137, Taiwan; 3 National Synchrotron Radiation Research Center, Hsinchu 30010, Taiwan; 4 Department of Applied Physics, National University of Kaohsiung, Kaohsiung 811726, Taiwan

**Keywords:** bismuth vanadate, photoelectrochemical water splitting, X-ray absorption spectroscopy, localized surface plasmon resonance

## Abstract

Bismuth vanadate (BiVO_4_) has attracted substantial attention on account of its usefulness in producing hydrogen by photoelectrochemical (PEC) water splitting. The exploitation of BiVO_4_ for this purpose is yet limited by severe charge recombination in the bulk of BiVO_4_, which is caused by the short diffusion length of the photoexcited charge carriers and inefficient charge separation. Enormous effort has been made to improve the photocurrent density and solar-to-hydrogen conversion efficiency of BiVO_4_. This study demonstrates that modulating the composition of the electrode and the electronic configuration of BiVO_4_ by decoration with silver nanoparticles (Ag NPs) is effective in not only enhancing the charge carrier concentration but also suppressing charge recombination in the solar water splitting process. Decoration with a small number of Ag NPs significantly enhances the photocurrent density of BiVO_4_ to an extent that increases with the concentration of the Ag NPs. At 0.5% Ag NPs, the photocurrent density approaches 4.1 mA cm^−2^ at 1.23 V versus a reversible hydrogen electrode (RHE) under solar simulated light illumination; this value is much higher than the 2.3 mA cm^−2^ of pure BiVO_4_ under the same conditions. X-ray absorption spectroscopy (XAS) is utilized to investigate the electronic structure of pure BiVO_4_ and its modification by decoration with Ag NPs. Analytical results indicate that increased distortion of the VO_4_ tetrahedra alters the V 3d–O 2p hybridized states. Additionally, as the Ag concentration increases, the oxygen vacancy defects that act as recombination centers in BiVO_4_ are reduced. In situ XAS, which is conducted under dark and solar illumination conditions, reveals that the significantly enhanced PEC performance is attributable to the synergy of modulated atomic/electronic structures and the localized surface plasmon resonance effect of the Ag nanoparticles.

## 1. Introduction

The development and use of solar energy to solve urgent energy and environmental problems have become the tasks of top priority in the last few decades owing to the rapid depletion of fossil fuels. Artificial photosynthesis, which can directly convert solar energy into hydrogen and other fuels, has been an efficient means of meeting some of the rising global energy demand [1]. Hydrogen is a storable, environmentally friendly fuel that is straightforwardly combusted in fuel cells to generate an electric current and heat without any harmful by-products [2]. After its discovery by Honda and Fujishima in 1972 [3], PEC water splitting using semiconductors as photoelectrodes has become a promising means of hydrogen production. TiO_2_ [4], Fe_2_O_3_ [5], WO_3_ [6] and ZnO [7] are typical semiconducting materials with superior photocatalytic ability. However, their large bandgap, low charge carrier mobility, high electron–hole recombination rate and slow kinetics during oxygen evolution reaction (OER) reduce their solar conversion efficiency. An ideal photoanode with excellent light harvesting ability and the efficient separation and transport of photogenerated charge carriers is sought for highly efficient water splitting. As a semiconductor widely used in photocatalytic applications, monoclinic bismuth vanadate (m-BiVO_4_) with a direct bandgap of about 2.4 eV has emerged as a promising candidate catalyst for use in hydrogen production by PEC water splitting. Thanks to its favorable band edge positions, low environmental toxicity and high aqueous stability, BiVO_4_ clearly has the potential to become a highly efficient PEC photocatalyst [8]. The theoretical solar-to-hydrogen conversion efficiency of BiVO_4_ approaches 9.2% with a maximum photocurrent density of 7.5 mA cm^−2^ under Xe lamp solar simulated light illumination [9]. However, BiVO_4_ underperforms compared to theoretical calculations. Recent reports have indicated that the severe recombination of photogenerated electron–hole pairs in the bulk, and on the surface, of BiVO_4_ are the main factors that limit its PEC performance. Some strategies such as elemental doping [10], the formation of heterojunctions [11] and coupling with different oxygen evolution catalysts (OECs) [12] have been proposed to enhance the water splitting efficiency of BiVO_4_. 

The field of plasmonic metal nanoparticles has expanded rapidly because of their ability to confine light in the vicinity of their surface and their strong surface plasmon resonance (SPR) [8,13,14,15]. While nanostructured gold is most commonly used in plasmonic applications, its replacement with silver is highly desirable because nanostructured silver reduces optical loss [16] and exhibits intense, localized surface plasmon resonance (LSPR) in the visible spectrum. Some studies have suggested that metal nanoparticles enhance solar water splitting by trapping light, direct electron transfer (DET) and plasmon resonance electron transfer (PRET) from the metal to the semiconductor. While DET produces hot electrons that are injected directly into the conduction band of the semiconductor, PRET increases the intensity of the electric field on the surface of the semiconductor, increasing photon absorption near the surface and allowing the formation of electron–hole pairs in its near-surface region [17]. Both DET and PRET mechanisms from LSPR significantly improve the photoactivity of the photoanode in PEC water spitting by inhibiting charge carrier recombination. Moreover, when noble metals come into contact with semiconductors, a Schottky barrier is formed as a result of their different work functions, so the electrons at the interface flow to the noble metal, establishing the built-in electric field from the semiconductor to the metal. This phenomenon induces electron rectification at the metal–semiconductor interface, promoting charge separation, improving charge transfer and modifying the electronic band structure [18], which can enhance PEC performance.

Many studies of the combination of BiVO_4_ with plasmonic Ag NPs have been published lately. Patil et al. showed that the ternary BiVO_4_/Ag/rGO hybrid photoanode with 2% Ag NPs enhanced PEC performance owing to the synergetic effects of the Schottky barrier formation, localized surface plasmon resonance and excellent electrical properties [19]. Tayebi et al. estimated key PEC parameters and found that the 2% Ag–BiVO_4_ photoanode exhibited a greater improvement in PEC performance than other tested photoanodes owing to the effect of the incorporated Ag dopant [20]. Jeong et al. developed a nanocomposite photoanode that was based on an Ag-NPs-impregnated BiVO_4_ film, which exhibited remarkably enhanced photocurrent density owing to improved carrier generation and charge separation as a result of LSPR [21]. Interface charge separation and transportation can be improved by the evolution of the induced internal electric field and defect moderation in the interface region. In the presence of Ag NPs, plasmonic oscillation can change the electronic structure of the semiconductor by modifying the plasmonic metal/semiconductor interface states and generating localized transition states. Although the aforementioned characteristics of BiVO_4_ and plasmonic Ag NPs have been intensively studied, to the best of our knowledge, the detailed mechanism of the photocatalytic reaction under solar irradiation and the influence of Ag NPs on BiVO_4_ electronic structure remain unclear. We believe that understanding the mechanism of the photocatalytic reaction of the host semiconductor is critical for the future exploitation of plasmonic PEC applications.

To shed light on the plasmonic effect on PEC performance, BiVO_4_ photoanodes are decorated with different concentrations of Ag NPs (0.0%, 0.1%, 0.3% and 0.5%). The structural properties, photoactivity and PEC efficiency of photoanodes are analyzed. X-ray absorption spectroscopy (XAS) is carried out to determine the electronic structure of pure BiVO_4_ and its modulation by decoration with plasmonic Ag NPs. In situ XAS under illumination is used to confirm the evolution of the localized plasmon resonance effect, revealing the dominant mechanism of the enhanced PEC performance. The relationships between Ag-decorated concentration and both electronic structure modification and PEC performance are comprehensively discussed.

## 2. Materials and Methods

### 2.1. Synthesis of Pure BiVO_4_ and Ag–BiVO_4_

**Scheme 1:**
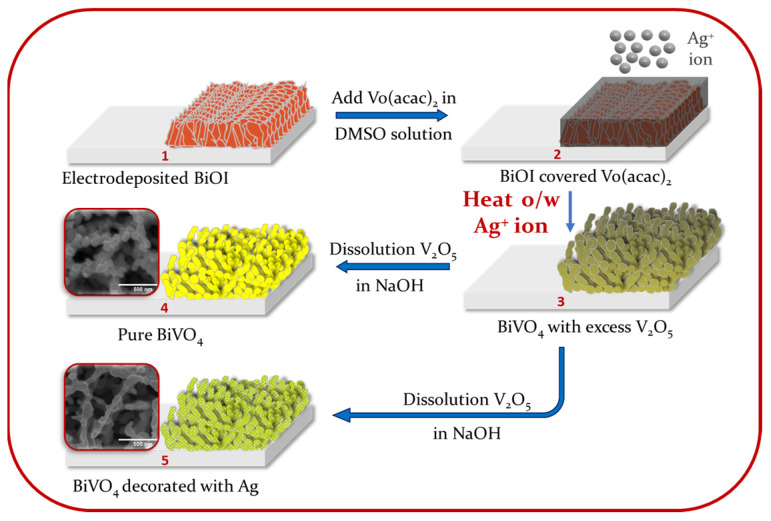
Synthesis of pure BiVO_4_ and Ag–BiVO_4_ by two-step method of electrodeposition.

Pure BiVO_4_ and Ag–BiVO_4_ films are prepared by the two-step method of the electrodeposition of bismuth oxyiodide (BiOI) on fluorine-doped tin oxide (FTO) glass substrate and the thermal treatment of BiOI precursor film with a solution of vanadyl acetylacetonate VO(acac)_2_ and AgNO_3_ precursors at an appropriate temperature. The BiOI film is prepared by the electrodeposition method, as described elsewhere. [22] Subsequently, VO(acac)_2_ solution in dimethyl sulfoxide (DMSO) that contains a suitable amount of AgNO_3_ is dropped onto the surface of the resultant BiOI film (1 × 1 cm^2^). The concentration of AgNO_3_ is estimated to range from 0.1% to 0.5% with various molar content of VO(acac)_2_. Then, the obtained samples are annealed under the air condition at 100 °C in a furnace for 60 min and subsequently heated up to 400 °C for another 60 min. The preparation method herein results in the formation of a homogenous BiVO_4_ surface. Through the annealing, nanoflaky BiOI precursors are transformed into a nanoporous BiVO_4_ structure. Following thermal treatment, the electrodes are soaked in NaOH solution to remove excess V_2_O_5_ from their surfaces. A pure BiVO_4_ sample without Ag decoration is also prepared for comparison. Scheme 1 displays the synthesis of pure BiVO_4_ and Ag–BiVO_4_ below.

### 2.2. Photoelectrochemical Measurement

To obtain photoelectrochemical performance, BiVO_4_ samples (1 × 1 cm^2^), Pt wire and Ag/AgCl 1 M KCl are employed as the working photoanode, counter electrode and reference electrode, respectively. The photoelectrochemical measurements are carried out in a pH 7 phosphate buffer (0.1 M KPi) electrolyte solution by using a 150 W Xe lamp solar simulator. The light intensity shone at the working electrode through back irradiation is about 100 mW/cm^2^. The scan rate is set at 10 mV/s for the photoelectrochemical measurement in the dark and under light illumination. The acquired potential against the Ag/AgCl reference electrode is then converted to that versus a reversible hydrogen electrode (RHE) using the follow equation:

V_RHE_ = V_Ag/AgCl_ + 0.197 + 0.059 × pH

### 2.3. Characterization

The morphological features of the as-prepared samples are identified using a scanning electron microscope (FE-SEM, Model Hitachi S4800). The phase determination of the sample is carried out using X-ray diffraction (XRD). The microstructure is determined by high-resolution transmission electron microscopy (HR-TEM). The Raman spectra are recorded using a micro-Raman spectrometer (LabRAM HR800 Horiba Jobin Yvon) that is operated with a wavelength of 532 nm. Optical absorption spectra are obtained using a UV–vis spectrometer (U-3900 spectrophotometer). 

X-ray absorption measurements are performed at the National Synchrotron Radiation Research Center (NSRRC), Taiwan. XAS spectra of V K-edge are measured at Taiwan Light Source (TLS) BL-17C using fluorescent mode at room temperature with an energy resolution of 0.25 eV. V K-edge has an absorption energy of 5465 eV, and it is bulk sensitive with a probing depth of about 100 nm. Ag K-edge is carried out in fluorescence mode at Taiwan Photon Source (TPS) BL-44A, the energy resolution of which is 1.25 eV. The V L-edge and O K-edge spectra are measured at TLS BL-20A (energy resolution of 0.05eV) in total electron yield mode, which is sensitive to the surface with 5–10 nm probing depth. The X-ray-excited optical luminescence and X-ray absorption–emission (XES-XAS) spectra at O K-edge are obtained, respectively, at beamlines BL23A and BL45A2 TKU end-station at TPS. The O K-edge XES spectra are collected using a high-resolution emission spectrometer with variable line spacing (VLS) grating spectrometer (high-density grating 18000 lines/cm) that has resolving power of about 5000 at O K-edge.

## 3. Results and Discussion

**Figure 1:**
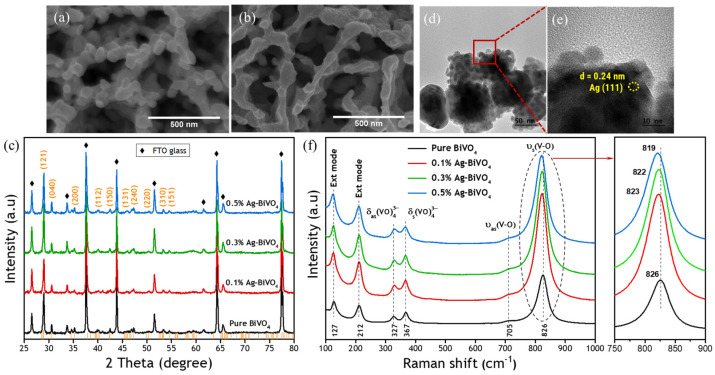
SEM images of surfaces of (**a**) bare BiVO_4_ and (**b**) 0.5% Ag–BiVO_4_; (**c**) XRD patterns of same with indicated FTO glass signals. Orange, vertical lines are standard peaks of monoclinic BiVO_4_ (JCPDS 00−14−0688); TEM images of 0.5% Ag–BiVO_4_ at different magnifications: (**d**) 50 nm and (**e**) 10 nm scale bar. (**f**) Raman spectra of bare BiVO_4_ and Ag–BiVO_4_ samples with assigned stretching and bending modes, respectively annotated.

Figure 1a,b presents top-view SEM images of the morphologies of pure BiVO_4_ and Ag–BiVO_4_ photoanodes. The pure BiVO_4_ and Ag–BiVO_4_ samples have similar, nanoporous morphologies with vertically aligned BiVO_4_ nanoplates on FTO glass substrates. The surface of the Ag–BiVO_4_ sample has fairly clear traces of nanosized Ag as its surface is rougher than that of pure BiVO_4_. XRD analysis is performed to determine phase purity and crystalline structure, as shown in Figure 1c. The XRD patterns reveal the monoclinic phase of BiVO_4_ crystal with the scheelite structure, consistent with data in the literature (JCPDS no. 00-14-0688) [23]. The absence of any obvious peak of silver in the samples that are decorated with Ag may be due to either the much weaker diffraction intensities of Ag than those of pure BiVO_4_ or the very low Ag content. A TEM investigation is subsequently performed not only to determine the fine crystal structure but also to obtain an accurate view of the Ag NPs on the BiVO_4_ films. The high-resolution TEM (HRTEM) image of 0.5% Ag–BiVO_4_ in Figure 1d suggests that the interplanar distance is 0.24 nm (Figure 1e), which corresponds to the wurtzite structure of Ag with (111) as the crystallographic plane [24]. Notably, the possible existence of AgOx cannot be completely ruled out, as minor AgO_x_ (Ag_2_O and AgO) can be traced by high-resolution TEM and fast Fourier-transform analysis [25]. TEM is a local probe, and the EDS elemental mapping also indicates that the Ag appears only on a certain area on the BiVO_4_ surface. Consequently, the close of oxygen vacancies could also be associated with the surface AgO_x_ species that form V–O–Ag on the surface of BiVO_4_, which is addressed in a later section. 

Raman scattering spectroscopy is an efficient tool for determining the structure and bonding of metal oxide species from their vibrational characteristics. Therefore, Raman spectra are obtained here to determine the phase/structure of pure and Ag–BiVO_4_, as shown in Figure 1f. All Raman spectral features are consistent with monoclinic BiVO_4_, and six noticeable vibrational bands are clearly obtained at 127, 212, 327, 367, 710 and 826 cm^−1^. The peaks at 127 and 212 cm^−1^ originate from external modes that correspond to vibration of the crystal lattice. Distinctive antisymmetric (δ_as_) and symmetric (δ_s_) bending modes appear at about 327 and 367 cm^−1^, respectively. The signal at 367 cm^−1^ is stronger than that at 327 cm^−1^ because the symmetric default is more than the antisymmetric default in the VO_4_ unit [26]. Remarkably, the relative intensity δ_s_/δ_as_ decreases as the Ag concentration increases, suggesting a reduction in the symmetry in the VO_4_ unit. The most intense Raman band at 826 cm^−1^ is assigned to the symmetric V–O stretching mode, while the weak shoulder at 705 cm^−1^ is assigned to the asymmetric V–O stretching mode. The Ag decoration makes the Raman peak visibly more intense than that of the pure sample, possibly owing to the greater degree of BiVO_4_ crystallization, as Raman spectroscopy is sensitive to the surface structure. The high-intensity Raman band is slightly shifted to a lower frequency as a result of Ag decoration, from 826 cm^−1^ (pure BiVO_4_) to 819 cm^−1^ (0.5% Ag–BiVO_4_). The shift of the symmetric V–O stretching mode is attributable to the effect of Ag NPs on the vibrational mode of the V–O bond. The effect of oxygen vacancies in metal oxides for water splitting has been widely discussed in recent years. Previous studies have demonstrated that the oxygen vacancies are important for OER by modifying the surface properties (e.g., molecular adsorption and desorption) and bulk properties (e.g., energy level, electronic conductivity, charge carrier transportation) [27,28,29,30]. In addition, the oxygen vacancies can act as electron donors. The presence of oxygen vacancies can also possibly reduce the bandgap. The presence of oxygen vacancies can improve the electronic conductivity and enhance the charge transfer, favoring OER ability. [31,32] However, some reports demonstrated that the oxygen vacancies can act as recombination centers to limit the photocatalytic ability [33,34]. The drawbacks of BiVO_4_ for water splitting include low charge carrier mobility and high electron–hole recombination rate, which are strongly correlated with the oxygen vacancies. Thus, oxygen vacancies, which are crucially involved in water splitting reactions, can reportedly distort the BiVO_4_ crystal structure [35,36,37]. The decoration of Ag NPs is proposed to have an impact on the oxygen vacancy defects at the BiVO_4_ surface, which are expected to influence the water splitting reaction. 

**Figure 2:**
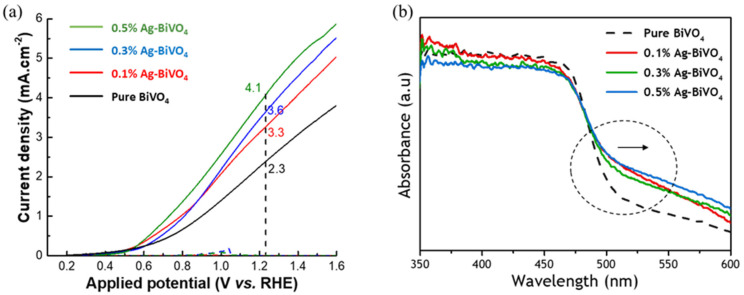
(**a**) Photocurrent densities of pure BiVO_4_ and Ag–BiVO_4_ photoanodes in the dark and under solar illumination. (**b**) UV–visible spectra of pure BiVO_4_ and Ag–BiVO_4_.

Figure 2a plots the measured photocurrent density versus potential (J-V) and linear sweep voltammetry (LSV) curves for BiVO_4_ electrodes. The photocurrent densities in the dark are negligible for all photoelectrodes even at high applied potential. Under illumination, the photocurrent density of pure BiVO_4_ increases linearly with the applied potential, reaching 2.3 mA cm^−2^ at 1.23 V vs. RHE. As the Ag concentration increases, the photocurrent density significantly increases, reaching 3.3 and 3.6 mA cm^−2^ in 0.1% and 0.3% Ag–BiVO_4_ photoanodes, respectively. The 0.5% Ag–BiVO_4_ photoanode yields the highest photocurrent density of 4.1 mA cm^−2^ at 1.23 V vs. RHE, which is almost 200% of that of pure BiVO_4_. The optical absorption performance is one of key factors that determines the photocatalytic activity and the success of the decoration of Ag NPs on BiVO_4_ photoanodes. Figure 2b shows the UV–visible absorption spectra of pure BiVO_4_ and Ag–BiVO_4_ samples. Whereas BiVO_4_ absorbs in the UV–visible region with a band that is centered at around 500 nm, the absorption edges of Ag–BiVO_4_ photoanodes are redshifted by different concentrations of Ag NPs. This finding confirms that the improvement of the response at wavelengths longer than the absorption edge of pure BiVO_4_ is the result of the localized surface plasmon resonance (LSPR) effect, indicating that Ag NPs contribute positively to the overall absorption of light by BiVO_4_ photoanodes. The hot electron injection that is induced by direct electron transfer (DET) has been previously proposed to be the main cause, rather than plasmon resonance electron transfer (PRET), of plasmon-assisted TiO_2_ water splitting [8,15,38] because TiO_2_ exhibits no inter-band excitation at wavelengths at which it would be enhanced by the plasmonic resonance of metal nanoparticles. Nevertheless, with respect to BiVO_4_ photoanodes, the inherent inter-band excitation of BiVO_4_ is responsible for weak hot electron injection because the absorption of visible light is induced by the LSPR effect in the absorption region of the host semiconductor [8]. Therefore, the PRET is proposed to be the main mechanism of the enhancement of the photocurrent density in Ag–BiVO_4_ photoanodes.

**Figure 3:**
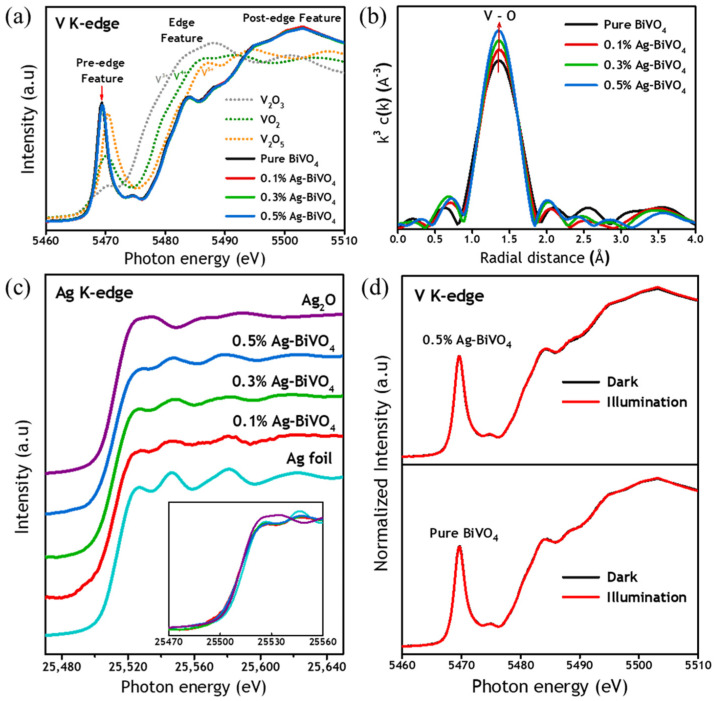
(**a**) XAS spectra at V K-edge. The pre-edge feature is assigned to 1s → 3d transition (allowed by 3d–4p orbital hybridization). Above the absorption edge, the first strong peak is assigned to a dipole-allowed transition 1s → 4p. (**b**) Fourier-transformed EXAFS of V K-edge in R-space. (**c**) XANES spectra at Ag K-edge. (**d**) In situ XAS at V K-edge in the dark and under illumination.

To determine the origin of the enhancement of PEC water splitting performance, X-ray absorption spectroscopy (XAS) is performed to elucidate the electron transfer efficiency of the photoanodes. Figure 3a compares the normalized X-ray absorption near-edge structure (XANES) spectra of BiVO_4_ and Ag–BiVO_4_ at the V K-edge with the reference spectra of V_2_O_3_, VO_2_ and V_2_O_5_. The spectra of pure and Ag-decorated BiVO_4_ have similar profiles, suggesting that decoration with Ag NPs does not alter the BiVO_4_ crystal structure. This result agrees with the aforementioned XRD and Raman results. The energy position of the absorption edges shows a good match with that of V_2_O_5_, indicating the preserved +5 oxidation states of vanadium in pure BiVO_4_ and Ag-decorated BiVO_4_. The extended X-ray absorption fine structure (EXAFS) spectra at the V K-edge are also investigated to study the local atomic environment. Figure 3b presents the Fourier-transformed amplitude of V K-edge EXAFS *k^3^χ* data, which show that the peak intensity increases with the concentration of decorative Ag NPs. The change in peak intensity typically arises from the variation of the coordination number of the metal center [39,40]. During the formation of oxygen vacancy defects, the lattice oxygen atoms escape from the structure [41], reducing the coordination number of the V atoms. In order to study the structural information in detail [42,43] (References [42,43] are cited in the supplementary materials), the EXAFS fitting results and parameters are displayed in Figure S1 (Supplementary Materials) and Table S1 (Supplementary Materials). The increase in the peak intensity of the V–O bond in R-space as a result of the decoration of Ag NPs on BiVO_4_ suggests an increase in the oxygen coordination number around the V atom centers. This finding reflects the reduction in the number of oxygen vacancy defects, which can act as recombination centers in BiVO_4_ that has been decorated with Ag NPs.

To confirm the oxidation state of Ag in our samples, the Ag K-edge is measured for all films that contain Ag NPs, as shown in Figure 3c. The absorption edges of these samples hardly vary. The Ag signals are closed to the characteristic spectrum of Ag foil, indicating that the Ag is in its metallic form in all Ag-decorated BiVO_4_ samples. Figure 3d shows V K-edge spectra of both pure and 0.5% Ag–BiVO_4_, which are found barely to differ between the dark and illumination conditions, indicating that V 4p states are not active sites for the PEC water splitting reaction. This finding can be understood with reference to the fact that the conduction band of BiVO_4_ is mainly composed of a hybridization of V 3d and O 2p states. Consequently, XAS at the O K-edge and V L-edge is used to determine the electronic structure of Ag NPs–BiVO_4_.

**Figure 4:**
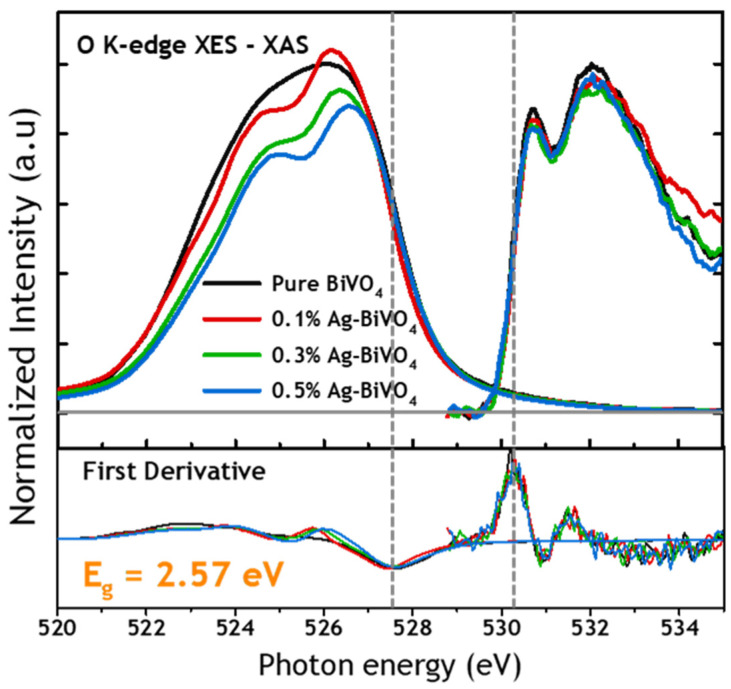
O K-edge XES-XAS spectra of pure BiVO_4_ and Ag–BiVO_4_. The first derivatives of all spectra are plotted underneath. Vertical lines indicate energy separation between two edges.

The probing of the O K-edge by X-ray emission spectroscopy (XES) and X-ray absorption spectroscopy (XAS) provides a direct means of estimating the energy difference between the highest occupied molecular orbital (HOMO) of the valence band and the lowest unoccupied molecular orbital (LUMO) of the conduction band. Figure 4 compares the absorption–emission (XAS-XES) spectra of pure and Ag-decorated BiVO_4_ and presents the corresponding first derivatives underneath. The zero crossing of the first derivatives of the XES-XAS spectra reveals the valence band maximum (VBM) and conduction band minimum (CBM). An energy bandgap of 2.57 eV is, therefore, obtained for both pure BiVO_4_ and Ag-decorated BiVO_4_ samples. This result is in agreement with a value previously reported [38,39,40]. Accordingly, loading with a small number of Ag NPs does not result in any significant change in the energy band gap of BiVO_4_. 

**Figure 5:**
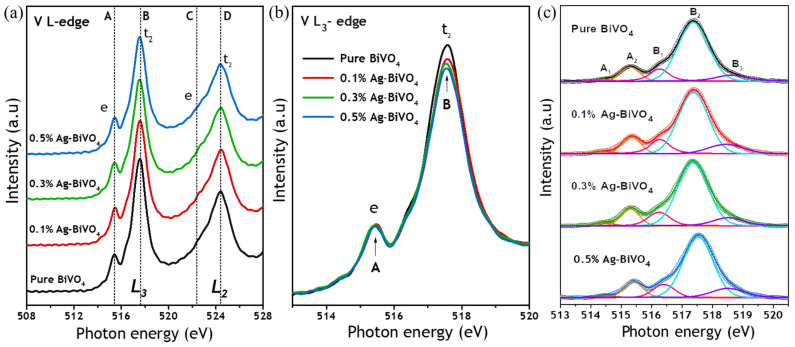
(**a**) V L-edge XAS spectra of pure BiVO_4_ and Ag–BiVO_4_. (**b**) Magnified view of the V L_3_-edge XAS spectra. (**c**) The resultant fit to the V L_3_-edge XAS spectra of the samples.

Figure 5 shows V L_2,3_-edge XAS spectra of pure BiVO_4_ and Ag–BiVO_4_ films. The spectra are dominated by two regions, which are L_3_ (excitation from 2p_3/2_ core levels to unoccupied CB V 3d states) and L_2_ (excitation from 2p_1/2_ core levels to unoccupied CB V 3d states). The two regions are separated by 6.65 eV due to the spin–orbit coupling of the 2p core–hole, consistent with the work of Cooper et al. [38,41]. There are five d-orbitals orientations, i.e., *3d_xy_*, *3d_xz_*, *3d_yz_*, *3d_z2_* and *3d_x2−y2_*, under VO_4_ tetrahedral symmetry. The *3d_x2−y2_* orbital mainly distributes along the z-axis, and the *3d_x2−y2_* orbital is directed along the x- and y-axis. The high-energy *3d_xy_*, *3d_xz_* and *3d_yz_* states have similar orbital symmetry, but lie in the xy, xz and yz planes. Thus, the tetrahedral VO_4_ is strongly related to the t_2_ orbitals. The V L_3_-edge spectra (Figure 5a,b) include a peak at high energy (517.5 eV), originating from 3d t_2_ (*3d_xy_*, *3d_xz_*, *3d_yz_*) states and associated with local atomic symmetry, which is lower for Ag–BiVO_4_ than for pure BiVO_4_. The change in intensity of this peak may be attributable to a change in (1) the number of unoccupied states or (2) local atomic symmetry [44]. To investigate the local atomic symmetry, the spectra are deconvoluted into five components (peaks A_1,_ A_2_, B_1_, B_2_ and B_3_ in Figure 5c) [38,41]. If the change of peak intensity arises from the changes in unoccupied electronic states, the relative areas of peak B_1_, B_2_ and B_3_ in Ag–BiVO_4_ should be similar to those in pure BiVO_4_. However, after the fitting of the five components for all the samples, estimating the ratios of the areas of peak B_1_, B_2_ and B_3_ reveals a variation of the ratios between pure and Ag–BiVO_4_, which is clear evidence of a distorted tetrahedral environment around the V atoms in the BiVO_4_. 

**Figure 6:**
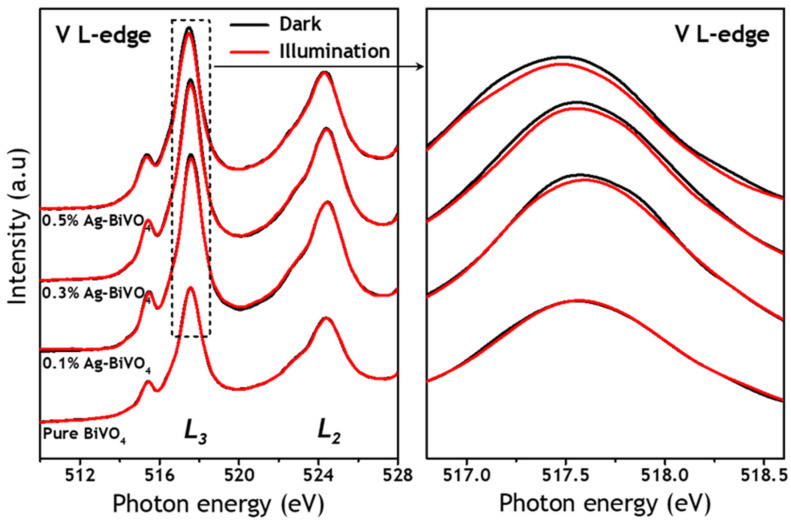
In situ XAS at V L-edge of the samples in the dark and under solar illumination.

To determine the origin of PEC enhancement and its underlying mechanism, in situ XAS spectra of the V L-edge are obtained in the dark and under illumination for all the films, as shown in Figure 6. With respect to pure BiVO_4_, the absorption intensity of the V L-edge does not detectably differ between the two conditions, suggesting that the photoinduced electron occupancy during illumination in the empty V 3d states is insignificant, probably owing to inherently rapid electron–hole recombination. However, the intensity of the V L_3_-edge under illumination is slightly reduced in the 0.1% Ag–BiVO_4_ photoanode, implying that unoccupied V 3d states gain some charges and start facilitating electron transfer. The result suggests that decoration with Ag NPs can generate more efficient, photoexcited electrons and holes in BiVO_4_ by the localized surface plasmon resonance effect. Likewise, under illumination, the intensity of the V L_3_-edge of 0.3% Ag–BiVO_4_ decreases, and the difference in intensity between conditions with and without illumination is greater for 0.3% Ag–BiVO_4_ than for 0.1% Ag–BiVO_4_, revealing more efficient generation of photoexcited electrons and holes. Ultimately, the difference in the absorption intensity between the dark and illumination conditions is greatest for the 0.5% Ag–BiVO_4_ photoanode because it exhibits the strongest LSPR effect that is induced by the Ag NPs. This result is consistent with its PEC performance.

**Figure 7:**
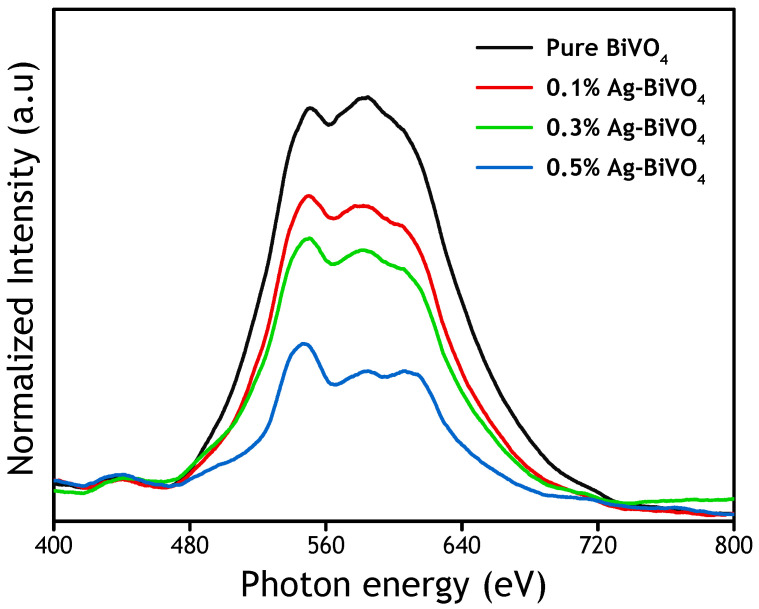
X-ray-excited optical luminescence (XEOL) of BiVO_4_ with and without decoration with Ag NPs.

X-ray-excited optical luminescence (XEOL) is used to monitor optical luminescence (UV–visible–NIR) that is excited at a desired excitation photon energy. In this technique, a particular core electron of a given element is excited to bound, quasi-bound and continuum states, providing element and site specificity [45,46]. Figure 7 displays XEOL spectra that are recorded with an excitation energy above the V K-edge. Each spectrum comprises two broad bands that peak around 550 nm and 580 nm. The main emission peaks of all the samples are at approximately 550 nm, implying the recombination of band-to-band radiative transitions. Decoration with Ag NPs significantly changes the luminescence spectra; it changes not only the total spectra intensity but also the emission features of the second broad band which is located approximately at 580 nm. The decrease in emission intensity indicates that the electron–hole recombination in BiVO_4_ can be effectively suppressed, facilitating the displacement of holes closer to the surface, favoring the water oxidation reaction and enhancing PEC performance. The intensities of the emission features ca. 580 nm that are considered to be involved in emission by defects in BiVO_4_ decrease as the Ag decoration concentration increases. Liu et al. found that the loading of noble metal Pd nanoparticles on TiO_2_ nanotubes changed their luminescence, in which the Pd plays two crucial roles—modifying the defect states on the surface of TiO_2_ and quenching the radiative recombination in TiO_2_ by the sinking of electrons [47]. Notably, the ratio of the intensities of these two broad bands changes appreciably, indicating that the moderation of defects in the BiVO_4_ crystal structure by decoration with Ag NPs strongly affects its luminescence properties. As revealed by the XEOL spectra in Figure 7, 0.5% Ag–BiVO_4_ film emits the least intensely as a result of both extrinsic radiative transition and defect emission. It is suggested that oxygen vacancy defects on the BiVO_4_ surface that act as the recombination center partially reduce upon decorating with Ag NPs.

**Figure 8:**
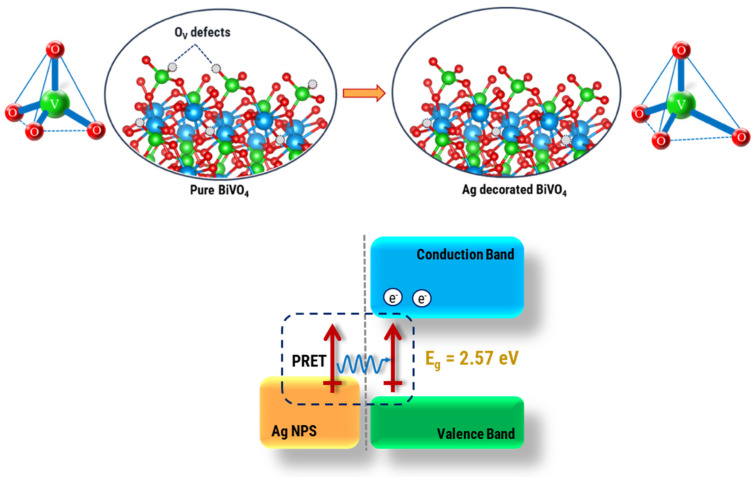
Schematic representative results concerning the effect of Ag NPs decorated on BiVO_4_.

Based on the above systematic investigations of the photoelectrochemical properties and the electronic structural evolution, Figure 8 presents representative results concerning the plasmonic effect caused by Ag NPs that are decorated on BiVO_4_ in this study. Analytical results indicate that the enhancement of distortion in the VO_4_ tetrahedra modifies the V 3d–O 2p hybridized states which resulted from the change of the number of oxygen defects. Additionally, increasing the Ag concentration reduces the concentration of oxygen vacancy defects that act as recombination centers in BiVO_4_. In situ XAS, conducted in the dark and under solar illumination, reveals significantly enhanced PEC performance that is attributable to the localized surface plasmon resonance effect of nanosized Ag particles. The BiVO_4_ surface is enriched with Ag NPs which modify it by the regulation of the oxygen vacancy defects, thereby improving the photoelectrochemical water splitting efficiency of the BiVO_4_. 

## 4. Conclusions

The decoration of BiVO_4_ photoanodes with Ag NPs modifies the local atomic and electronic structures of the host semiconductor by increasing the distortion of the VO_4_ tetrahedra. As the concentration of Ag NPs increases, the oxygen vacancy defects that act as recombination centers that are unfavorable to water oxidation in PEC water splitting are reduced. The localized surface plasmon resonance effect that is induced by nanosized Ag particles extends the light absorption range and increases the separation and transfer efficiency of photogenerated charge carriers. The relationship between PEC performance and Ag NPs decoration proportion is revealed, and the highest photocatalytic efficiency is 4.1 mA cm^−2^, reached at an Ag concentration of 0.5%. This work improves our understanding of the effect of electronic structure on photocatalytic activity and, thus, paves the way toward the design of high-performance semiconductor photoelectrodes for efficient solar energy conversion.

## Data Availability

The data is available on reasonable request from the corresponding author.

## Supplementary Materials

The following supporting information can be downloaded at https://www.mdpi.com/article/10.3390/nano12203659/s1, Figure S1: The fitting of EXAFS data. Two types of V-O bonds are used according to the scheelite monoclinic structure [1,2] and the analytical result indicates that the CN increases as Ag concentration increases; Table S1: EXAFS parameters. References [42,43] are cited in the supplementary materials.
