# Supplementary material for: Effect of Ag-Decorated BiVO4 on Photoelectrochemical Water Splitting: An X-ray Absorption Spectroscopic Investigation
Source: Nanomaterials (Basel). 2022 Oct 18;12(20):3659. doi: 10.3390/nano12203659 (PMC9609331; doi:10.3390/nano12203659)
Supplement: Supplementary file 1 [file nanomaterials-12-03659-s001.zip › nanomaterials-1937226-supplementary.pdf]

nanomaterials

MDPI

Supplementary information

Effect of Ag-Decorated BiVO4 on Photoelectrochemical Water Splitting: An X-ray Absorption Spectroscopic Investigation

Ta Thi Thuy Nga [1,2], Yu-Cheng Huang [1,2], Jeng-Lung Chen [3], Chi-Liang Chen [3], Bi-Hsuan Lin [3], Ping-Hung Yeh [2], Chao-Hung Du [2], Jau-Wern Chiou [4], Way-Faung Pong [2], K. Thanigai Arul [2], Chung-Li Dong [2,*] and Wu-Ching Chou [1,*]

[1] Department of Electrophysics, National Yang Ming Chiao Tung University, Hsinchu 30010, Taiwan
[2] Research Center for X-ray Science & Department of Physics, Tamkang University, New Taipei City 25137, Taiwan
[3] National Synchrotron Radiation Research Center, Hsinchu 30010, Taiwan
[4] Department of Applied Physics, National University of Kaohsiung, Kaohsiung 811726, Taiwan
* Correspondence: cldong@mail.tku.edu.tw (C.-L.D.); wcchou957@nycu.edu.tw (W.-C.C.)

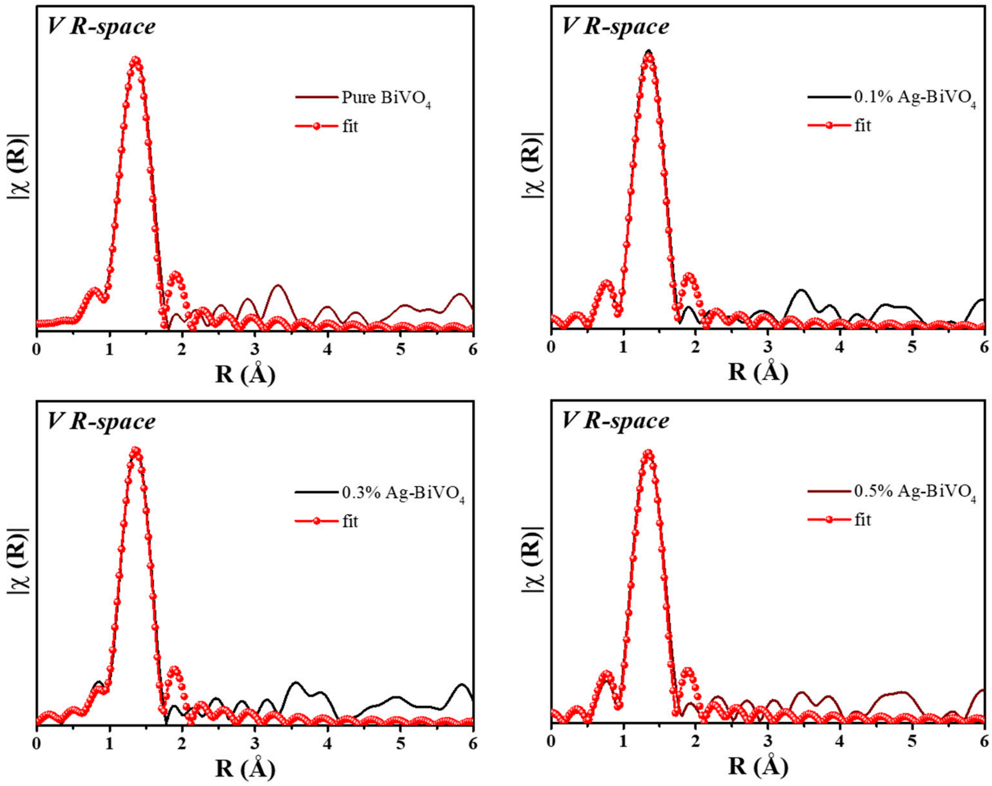

Figure S1. The fitting of EXAFS data. Two types of V-O bonds are used according to the scheelite monoclinic structure [1,2] and the analytical result indicates that the CN increases as Ag concentration increases.

Table S1. EXAFS parameters.

Sample	Bond type	CN	$\sigma^2 \times 10^{-3}$ (Å^{-2})	ΔE_0 (eV)	R (Å)	R -factor
**Pure $BiVO_4$**	V-O_1	1.67	2	10.37	1.699	0.03
	V-O_2	1.67	0.2	7.64	1.758	
**0.1% $BiVO_4$**	V-O_1	1.78	0.41	12.70	1.696	0.02
	V-O_2	1.78	0.53	4.17	1.758	
**0.3% $BiVO_4$**	V-O_1	1.86	1.69	12.89	1.706	0.03
	V-O_2	1.86	0.5	3.62	1.751	
**0.5% $BiVO_4$**	V-O_1	1.97	1.5	11.62	1.690	0.03
	V-O_2	1.97	0.32	2.81	1.754	

CN: coordination number. ΔE_0 (eV): inner potential correction to account for the difference in the inner potential between the sample and the reference compound. σ^2(Å^{-2}): Debye-Waller factor. R(Å): distance between absorber and backscatter atoms.

Reference

[1] Pattengale, B.; Ludwig, J.; Huang, J. Atomic insight into the W-doping effect on carrier dynamics and photoelectrochemical properties of BiVO4. *J. Phys. Chem. C* **2016,** *120*, 1421-1427

[2] Ding, K.; Chen, B.; Fang, Z.; Zhang, Y. Density functional theory study on the electronic and optical properties of three crystalline phases of $BiVO_4$. *Theor. Chem. Acc.* **2013**, *132*, 1352
